# Differences in Abnormal Water Metabolism between SD Rats and KM Mice Intoxicated by Microcystin-RR

**DOI:** 10.3390/ijerph18041900

**Published:** 2021-02-16

**Authors:** Wenqing Xiao, Qing Zhong, Feng Sun, Weiguang Wang, Zhiyao Zhao, Kangding Gu

**Affiliations:** MOE Key Lab of Environment and Health, Institute of Environmental Medicine, School of Public Health, Tongji Medical College, Huazhong University of Science and Technology, Wuhan 430030, China; wenqing_xiao@126.com (W.X.); zhongqing0622@126.com (Q.Z.); sunfendoh@163.com (F.S.); wwg252621@163.com (W.W.); zzy-wh@126.com (Z.Z.)

**Keywords:** microcystin-RR, water metabolism, polydipsia, polyuria, nephrotoxicity

## Abstract

The effects of microcystin-RR (MC-RR) on water metabolism were studied on Sprague–Dawley (SD) rats and KunMing (KM) mice. In the single dose toxicity test, polydipsia, polyuria, hematuria and proteinuria were found in group of rats receiving a MC-RR dose of 574.7 μg/kg, and could be relieved by dexamethasone (DXM). Gradient damage was observed in kidney and liver in rats with gradient MC-RR doses of 574.7, 287.3, and 143.7 μg/kg. No significant water metabolic changes or kidney injuries were observed in mice treated with MC-RR doses of 210.0, 105.0, and 52.5 μg/kg. In the continuous exposure test, in which mice were administrated with 140.0, 70.0, and 35.0 μg/kg MC-RR for 28 days, mice in the 140.0 μg/kg group presented increasing polydipsia, polyuria, and liver damage. However, no anatomic or histological changes, including related serological and urinary indices, were found in the kidney. In summary, abnormal water metabolism can be induced by MC-RR in rats through kidney injury in single dose exposure; the kidney of SD rats is more sensitive to MC-RR than that of KM mouse; and polydipsia and polyuria in mice exposed to MC-RR for 28 days occurred but could not be attributed to kidney damage.

## 1. Introduction

Microcystins (MCs) are a group of natural cyclic peptides produced by multiple cyanobacterial species, with more than 279 analogues found so far [1,2], of which MC-LR and MC-RR are most common toxins [3,4,5,6]. According to published reports, MCs are specific hepatotoxic agents capable of causing acute liver damage in wild and domestic animals exposed to high doses and play a role in tumor promotion in long-term tested animals exposed to lower doses of these toxins [7,8,9,10,11]. To date, a large number of studies have investigated hepatic toxicity resulting from MCs [12,13,14,15]. Some studies have also reported nephrotoxicity, reproductive toxicity, and gastrointestinal toxicity caused by MCs in rats, fish and other species [1,16,17,18,19,20,21,22,23]. Regarding nephrotoxicity, whether the kidneys of different animals are all responsive to MCs is unclear.

Rats and mice are common animal models for toxicological studies in mammals. Previous studies reported consistent conclusions to support the nephrotoxicity of MCs in rats [19,20,24,25,26,27,28,29,30,31,32,33,34,35], but had different views on mice in terms of whether MCs could injure mouse kidneys. Qin, studying the toxicity of MC-LR in mice, found negative outcomes for the kidney, though they did not exclude possible endoplasmic reticulum stress (EMS) in kidney cells [26]. Jayaraj’s study reported that no inhibition of protein phosphatase activity was detected in the kidneys of mice under acute MC-LR exposure [27]. Al-Jassabi’s study suggested that MC-LR resulted in kidney tubule injury and renal oxidative damage in mice [28]. Xu’s research also suggested that MC-LR induced nephrotoxicity in mice [29]. In addition, Yi’s study reported histopathological changes in mouse kidney after three months of exposure to MC-LR [30]. However, most researchers focus on the tissue and cell injury and ignore the metabolic abnormalities. Due to water metabolism and electrolyte balance being tightly associated with kidney function [31], abnormal water metabolism is usually described as a symptom of kidney impairment and is often omitted when there are no obvious organ changes in the kidney. The effect of MCs on water metabolism in different test animals is unclear. Whether the changes of water intake and output in test animals induced by MCs are definitively caused by kidney damage also needs to be explored.

Over the past decades, most research has been focused on MC-LR and relatively little on MC-RR. These two analogues are cyclic heptapeptides that differ in one amino acid at two variable L-amino acid units (X and Y), MC-LR with a leucine and MC-RR with an arginine respectively [32]. This chemical differentiation results in differences in their nature and toxicity. Although the biotoxicity of MC-RR is a little bit lower than MC-LR [33,34,35], MC-RR is often detected in bodies of water and accounts for a higher proportion in the analogues. The level of MC-RR in these environments can be larger than MC-LR sometimes [36,37], so research on MC-RR for its toxicity is of great importance.

The present investigation aimed to understand the effect of MC-RR on water metabolism in the rat and mouse. Considering water metabolism is relevant to kidney function, the nephrotoxicity of MC-RR in the rat and mouse was also evaluated to explore the possible causes of water metabolic changes. As the liver is the main target of MCs and involves much metabolic reaction, some basic liver-related indices were monitored at the same time. This study was undertaken in two steps: a single dose toxicity test and a continuous exposure experiment, to explore and compare the effects of MC-RR on the two animals’ water metabolism and their related organ tropism.

## 2. Materials and Methods

### 2.1. Animals

Specific pathogen free (SPF) male Sprague–Dawley (SD) rats and KunMing (KM) mice were bought from the Laboratory Animal Center of Huazhong University of Science and Technology (Wuhan, China). All animals prior to the experiment were allowed to acclimatize to their surrounding conditions for a week with sufficient food and water ad libitum under a 12 h light/dark cycle and at 25 ± 2 °C room temperature.

### 2.2. MC-RR Extraction and Purification

MC-RR was extracted from Microcystis blooms occurring in Zhongshan Park, Wuhan (China). The extraction and purification procedures were as follows: 70% methanol aqueous solution was used to extract MCs from the collected cyanobacterial slurry with magnetic stirring at 120 r/min for 40 min at room temperature. The ratio of methanol aqueous solution (mL) to cyanobacterial slurry (g) was 10:1. The extracting solution was centrifuged at 9000× *g* for 8 min to collect the supernatant. To precipitate the phycocyanin, the supernatant was adjusted to pH 4 and placed stably for 120 min. Then, the phycocyanin was removed by centrifugation and filtration. After the phycocyanin was removed, the supernatant was adjusted to pH 7 and then methanol was removed by rotary evaporation. The crude extract was filtered through 0.45 μm pore size membrane filters to prepare for primary purification. Primary purification was performed by solid phase extraction (SPE) using an Oasis HLB column (Waters, Milford, MA, USA). The columns were activated by methanol and distilled water, and washed by 20 and 30% methanol aqueous solution successively after loading the crude extract. Then, 100% methanol was used to wash the columns for collection of the final eluent. The final eluent was concentrated to 2 mL by rotary evaporation for subsequent steps.

Secondary purification was performed by a Sephadex LH-20 column (500 mm × 26 mm i.d.) with 100% methanol eluted at 1 mL/min flow rate [38]. The eluent was collected every 5 mL per tube, and tubes that contained MC-RR (identified by high-performance liquid chromatography (HPLC)) were selected for the third purification steps. MC-RR was separated through the third purification process with a DEAE-52 (DEAE-cellulose) column (300 mm × 26 mm i.d.). In this process, distilled water and 3 mmol/L sodium solution eluted the column successively with a flow rate of 1 mL/min. The eluents were identified and quantified by HPLC according to the MC-RR standard (Enzo Life Sciences Inc., Lausen, Switzerland). Eluent with a purity of MC-RR > 90% was collected for use in the animal experiments (Supplementary Material Figure S1).

### 2.3. Experimental Procedures on Animals

#### 2.3.1. Single-Dose Toxicity Test

##### Single-Dose Toxicity Study of MC-RR in Rats and Mice for Water Metabolism

Rats aged 6 weeks and weighing 150–220 g were randomly divided into 4 groups, with 6 animals in each group. The animals were fed in metabolic cages and administered to MC-RR. According to the median lethal dose (LD_50_) obtained in the pre-experiment, the 4 groups of rats were given intraperitoneal injections of a high dose (574.7 μg/kg (3/4 LD_50_)), medium dose (287.3 μg/kg), low dose (143.7 μg/kg), or physiological saline as control. Mice aged 6 weeks and weighing 27–33 g were arranged in the same way as the rats, but the toxin MC-RR was administered at a high dose (210.0 μg/kg (3/4 LD_50_)), medium dose (105.0 μg/kg), low dose (52.5 μg/kg), with physiological saline as control. After 24 h, urine and blood samples of animal subjects were collected, and then the animals were euthanized and underwent postmortem and histopathologic examination. Weight gain, water intake, and urine output were recorded and calculated at two time periods: 24 h before and 24 h after intraperitoneal injection.

##### Blocking Tests of Rat Nephrotoxicity

The rats were separated into 5 groups. While being given the high doses of toxins described above, the rats were simultaneously administered different doses of dexamethasone (DXM, purity ≥97%, purchased from Sigma-Aldrich, St. Louis., MO, USA): high dose group DXM 50.0 mg/kg + MC-RR 574.7 μg/kg; medium dose group DXM 10.0 mg/kg + MC-RR 574.7 μg/kg; low dose group DXM 2.0 mg/kg + MC-RR 574.7 μg/kg; positive control group DXM 0.0 mg/kg + MC-RR 574.7 μg/kg, and saline control group. Twenty-four hours after administration, urine, blood and body organ samples were taken for biochemical and pathologic measurement and analysis to understand the changes in the structure and function of the kidney and liver.

#### 2.3.2. Continuous Exposure Experiment of MC-RR in Mice for Water Metabolism

Based on the results of single-dose toxicity of MC-RR in mice, a short-term toxicity test was performed to explore the possible impact on water metabolism and related organs. KM mice were randomly divided into 4 groups, with 35 in each group. The mice were fed in metabolic cages, with 7 in each cage in order to collect the urine effectively. The 4 groups were labeled in terms of toxin dosage given: high dose: 140.0 μg/kg; medium dose: 70.0 μg/kg; low dose: 35.0 μg/kg; and physiological saline as negative control. The animals were given intraperitoneal injection daily for 28 days, and continued to be observed until the 42nd day. The animals’ daily weight and drinking and urine volume were recorded. On the 7th, 14th, 28th, 35th, and 42nd days, 7 subjects in each group were sampled and euthanized for toxicological examination, including urinalysis, sodium and potassium ion levels in urine and serum, antidiuretic hormone (ADH) in blood, serum enzymes, kidney and liver weight, and histological examination.

#### 2.3.3. Sample Collection and Process

The urine volumes were pooled and recorded in each metabolic cage. The urine used for urinalysis, including specific gravity, Na^+^ and K^+^, was collected directly from the bladders. Blood samples were obtained from the optical orbits of the animals. The blood was naturally clotted and then centrifuged at 2500× *g* at 4 °C for 10 min to harvest the serum for following analysis. After blood collection was finished, the animals were euthanized to sample the organs, such as kidneys and liver. The organs were weighed and fixed in 4% polyformaldehyde for further histological examination.

### 2.4. Urinalysis

For routine urine and some chemical indices, semiquantitative colorimetric reagent strips (dry chemical method) (Acon, Hangzhou, China) were used for detection in urine. Urine specific gravity (USG) was determined using a LSUD-digital urinometer (Guangzhou Mingrui Electronic Technology Co., Ltd, Guangzhou, China).

### 2.5. Detection of Na^+^and K^+^ Levels in Urine and Blood

The urine and blood samples were diluted 200- and 100-fold, respectively, using deionized water prior to instrument detection for Na^+^ and K^+^ concentration. Samples were analyzed by an AA240Fs fast sequential atomic absorption spectrometer (Agilent, Santa Clara, CA, USA) coupled with flame atomization and sodium and potassium hollow cathode lamps with the current operating at 5 mA and air/C_2_H_2_ as gas form. The instrument working conditions were as follows: wavelength for sodium 589.6 nm, slit width 0.2 nm; wavelength for potassium 769.9 nm, slit width 1.0 nm.

### 2.6. Serum Biochemical Index Assay

A Mindray BS-200 Chemistry Analyzer (Shenzhen Mindray Bio-Medical Electronics Co., Ltd., Shenzhen, China) was applied for serum index detection, including albumin (ALB), alkaline phosphatase (ALP), alanine aminotransferase (ALT), glutamic oxaloacetic transaminase (aspartate aminotransferase, AST), creatinine (CREA), serum lactate dehydrogenase (LDH), glucose (Glu), total cholesterol (TC), total protein (TP), uric acid (UA), and urea nitrogen (BUN). ADH was measured using ELISA kits (Anoric Bio-technology Co., Ltd., Tianjin, China) according to the manufacturer’s instructions.

### 2.7. Histopathological Examination

The kidney and liver tissues fixed by polyformaldehyde were embedded in paraffin, cut to a thickness of 2 µm, stained with hematoxylin-eosin (HE), and examined using a light microscope. Periodic acid-Schiff (PAS) stain was applied to the kidney tissues to observe histological changes in glomerular mesangium, or basement membrane.

### 2.8. Statistical Analysis

Statistical analysis was performed with SPSS for Windows software version 19.0 (SPSS Inc, Chicago, IL, USA). The homogeneity of variances was tested using Levene’s test. One-way analysis of variance (ANOVA) was used for homogeneous data, followed by Dunnett’s test, and Dunnett’s T3 test was performed to compare the heterogeneous data. *p* < 0.05 was considered to be significant.

## 3. Results

### 3.1. Changes of Water Metabolism in Rats and Mice after Single-Dose Administration of MC-RR

#### 3.1.1. Water Intake, Urine Output

The urine output of the rats started to increase 6 h after administration of a high-dose of MC-RR (574.7 μg/kg). Gross hematuria appeared 12 h later and urine volume was significantly on the rise at 24 h. Other dosage groups did not show apparent alterations in urine output compared with the control group. Regarding water intake, no notable difference was observed in the rat groups except for a slight increase in the high-dose MC-RR group. As for the mouse subjects, water intake and urine excretion displayed similar volume levels between exposed groups and the control group (Table 1).

DXM had the effect of alleviating the abnormal output of urine. There was a dose-dependent effect of DXM in countering the symptoms of polyuria, in which the urine output of rats in the high dose group (DXM 50.0 mg/kg + MC-RR 574.7 μg/kg) almost approached the levels of the saline control group (Table 1).

#### 3.1.2. Behavior and Weight

Both rats and mice gradually went into a lethargic state after administration of high doses of MC-RR. Other symptoms included anorexia, cold limbs, etc. As seen in Table 1, the weight gain of the rats in the high-dose group sharply declined with statistical significance compared with the saline control group. Various amounts of DXM were able to reverse the weight loss of the rats in the high-dose group. The weight gain of rats in the DXM + MC-RR groups increased with the DXM dosage. Significant differences were shown in the weight gain of the groups given 10.0 and 50.0 mg/kg of DXM compared with the group given 574.7 μg/kg MC-RR (0.0 mg/kg DXM). Nevertheless, the body weights of the mice in each group did not change much, with no statistical difference between them.

#### 3.1.3. Urinalysis

Urinalysis showed a notable increase in urobilinogen, microalbumin, protein, and creatinine in the rats administered high-dose MC-RR. Occult blood and ketone were also detected, and the leukocyte count was much higher than that of the control group. Uric calcium and pH value were lower than normal ranges. Even in the medium-dose group of rats, urine protein displayed a rising trend accompanied by occult blood. However, no abnormal phenomena occurred in rats in the low-dose group. Under intervention with different levels of the DXM, all of the above indices (urobilinogen, microalbumin, protein, and creatinine) in the high-dose MC-RR groups tended to return to normal in a dose-effect manner, as shown in Table 2. In contrast, the urine indices for mice in any treated group showed no apparent differences with those of control.

The concentration of Na^+^ in urine in all groups of rats exposed to MC-RR were prominently higher than the control group (*p* = 0.000 for 574.7 μg/kg MC-RR group; *p* = 0.000 for 287.3 μg/kg MC-RR group; *p* = 0.001 for 143.7 μg/kg MC-RR group), showing a dose-response effect (Table 3). However, the levels of K^+^ in urine in all exposed groups of rats were lower than the control group (*p* = 0.000 for 574.7 μg/kg MC-RR group; *p* = 0.000 for 287.3 μg/kg MC-RR group; *p* = 0.003 for 143.7 μg/kg MC-RR group). On the other hand, the concentrations of Na^+^ and K^+^ in the urine of mice showed no differences between overall treated groups and the control group (*p* = 0.271 and 0.270, respectively).

From Table 4, it can be seen that the USG in rats in high dose group decreased slightly, while no statistical differences in USG have been observed between the test groups and the control groups either in rat or mice (rat *p* = 0.092; mouse *p* = 0.156).

#### 3.1.4. Serum Biochemical Indices in Rats and Mice in Single-Dose Toxicity Test

Serum biochemical analyses indicated that the UA in rats went down in high-dose group with statistical significance in comparison with the control in rats (*p* = 0.000). No significant differences were found in kidney indices of mice.

The ALT, AST, ALP, and LDH levels in rats in the high-dose group were significantly higher than those of the control group (*p* = 0.001, 0.000, 0.000, and 0.001, respectively). The ALT and AST of rats in the medium-dose group also increased compared with the control (*p* = 0.000). Regarding mice exposed to high-dose MC-RR, the ALT, AST, ALP, and LDH levels increased more than the control (ALT, AST, ALP *p* = 0.000; LDH *p* = 0.001). No changes in these indices in the low-dose group were observed.

The results indicate that a high dose of the toxin could cause damage to target organs such as the liver in both animals within 24 h. Using DXM to counter the toxicity could result in significantly lower serum indices (ALT, AST, ALP, and LDH) in tested animals in a dose-effect manner compared to the high-dose MC-RR group (Table 5).

Two inorganic elements in blood, Na^+^ or K^+^, were also measured in rats and mice. The Na^+^ in blood of rats in the high- and medium-dose groups was lower than the control (*p* = 0.000 and 0.022, respectively), whereas the K^+^ level in the high-dose group was higher than the control (*p* = 0.001). Such abnormal alterations in Na^+^ and K^+^ in the low-dose rat group and overall mouse groups were not observed (Table 3).

#### 3.1.5. Visceral Coefficients of Rats and Mice in Single-Dose Toxicity Test

There were some increases in the kidney/body and liver/body coefficients of the high-dose rat group, but there was no statistical significance compared with the control group. For rats in the medium- and low-dose groups and mice in all groups, no significant differences were observed in visceral coefficients in the detected organs (Table 4).

#### 3.1.6. Histological Examination of Rats and Mice in Single-Dose Experiment

**Figure 1:**
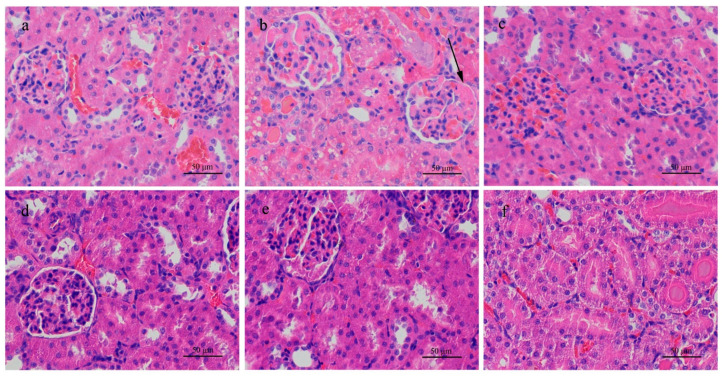
H & E stained histological sections of the kidneys of rats exposed to different concentrations of microcystin-RR (MC-RR) for 24 h. (**a**) 0.0 μg/kg MC-RR. (**b**) 574.7 μg/kg MC-RR. Necrosis of glomerular cells (arrow). (**c**) 287.3 μg/kg MC-RR. (**d**) 50.0 mg/kg DXM mixed with 574.7 μg/kg MC-RR. (**e**) 10.0 mg/kg DXM mixed with 574.7 μg/kg MC-RR. (**f**) 2.0 mg/kg DXM mixed with 574.7 μg/kg MC-RR.

**Figure 2:**
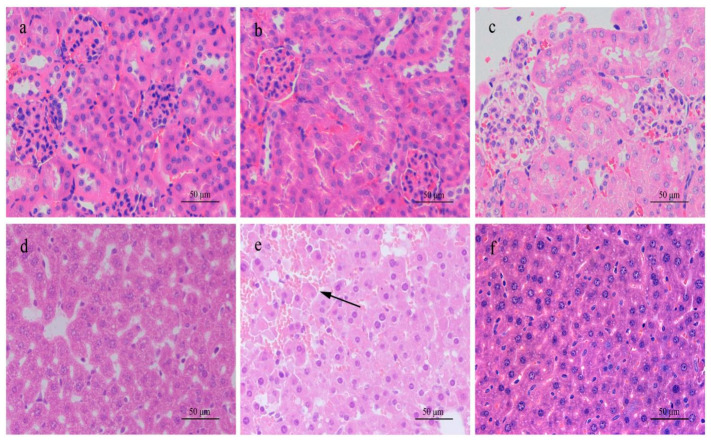
H & E stained histological sections of the kidneys and livers of mice exposed to different concentrations of MC-RR for 24 h. (**a**) Kidney in 0.0 μg/kg MC-RR group (control group). (**b**) Kidney in 210.0 μg/kg MC-RR group. (**c**) Kidney in 105.0 μg/kg MC-RR group. (**d**) Liver in 0.0 μg/kg MC-RR group (control group). (**e**) Liver in 210.0 μg/kg MC-RR group. Sinusoid expansion and hyperemia (arrow). (**f**) Liver in 105.0 μg/kg MC-RR group.

H&E stained sections of rat kidney from the high-dose group showed dilation of the kidney tubules, swollen or balloon-like endothelial cells, necrosis and detachment of cells, lymphatic cell infiltration, and destruction of normal kidney structures (Figure 1). In other toxin-treated groups of rats and all treated groups of mice, histopathological changes were not obvious compared with the control groups, as shown in Figure 1 and Figure 2. In the high-dose toxin experiments with intervention by different amounts of DXM, the low dose of DXM resulted in congestion of blood vessels, slight dilation of kidney tubules (Figure 1f), partial detachment of endothelial cells, and infiltration of inflammatory cells in rat kidney. With medium-dose DXM, infiltration of inflammatory cells could be seen, while with high-dose DXM, the kidney structures in rats were intact without necrosis, similar to the saline group, as shown in Figure 1.

**Figure 3:**
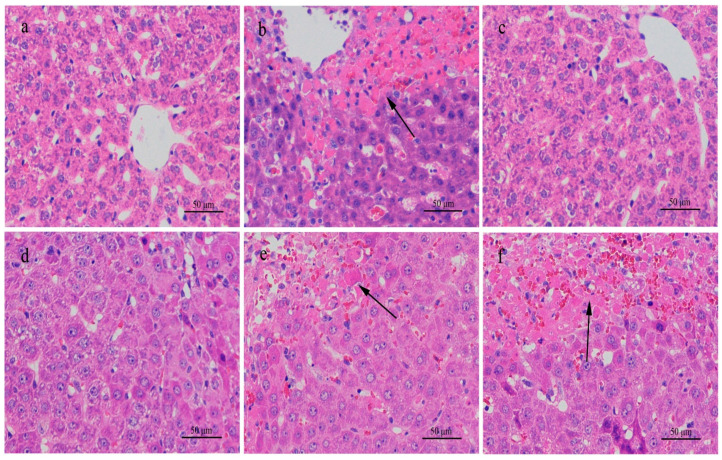
H & E stained histological sections of the livers of rats exposed to different concentrations of MC-RR for 24 h. (**a**) 0.0 μg/kg MC-RR. (**b**) 574.7 μg/kg MC-RR. (**c**) 287.3 μg/kg MC-RR. (**d**). 50.0 mg/kg DXM mixed with 574.7 μg/kg MC-RR. (**e**) 10.0 mg/kg DXM mixed with 574.7 μg/kg MC-RR. (**f**) 2.0 mg/kg DXM mixed with 574.7 μg/kg MC-RR. Note necrosis areas in Figure 3b,e,f (arrows).

Examined macroscopically, the rat liver in high-dose groups exhibited hyperemic swelling with some hemorrhagic spots. In histological sections stained with H&E, apparent dot necrosis of hepatocytes, swollen or balloon-like hepatocytes, infiltration of inflammatory cells, and sinusoidal dilatation occurred, but cord-like arrangements of hepatic cells remained in parts (Figure 3). Liver sections stained with H&E in the medium-dose rat group showed inflammable cell infiltration. However, no significant histopathological changes were observed for the low-dose group. In contrast, histological sections stained with H&E from mice in the high-dose group displayed an enlarged texture of the liver compared to the saline control group. Under an optical microscope, sinusoid expansion, cellular swelling, and infiltration of inflammatory cells could be seen, but the hepatic cord remained intact (Figure 2). In sections from mice given a medium dose, only inflammatory cell infiltration was observed. No abnormal changes were found in sections from mice given a low dose.

### 3.2. Changes of Water Metabolism in KM Mice under the Continuous Exposure to MC-RR

#### 3.2.1. Water Intake and Urine Output

**Figure 4:**
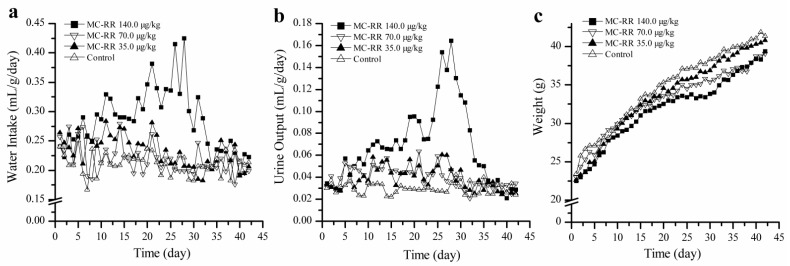
Water intake (**a**), urine output (**b**) and body weight (**c**) of mice treated with MC-RR in the continuous exposure experiment.

As shown in Figure 4a, the water intake of mice in the high-dose group rose on the ninth day and increased more quickly than that of other groups. The intake dropped abruptly on day 30 after the toxin was stopped, and returned to the average levels of the other groups until day 34. Mice in the medium- and low-dose groups did not show obvious differences in water intake compared with the physiological saline group.

The urine output in the high-dose group began to rise on day 8 and went up day-by-day until the toxin was stopped. Then the urine volume declined and was back to levels similar to the control group. Urine excretion in the other groups did not display significant alterations compared with the control, as seen in Figure 4b.

#### 3.2.2. Body Weight

The body weight gain of mice in the high-dose group started to decline clearly on day 9 and these mice continued to lose more than other groups until the toxin was stopped. After the toxin was stopped, the weight of the mice gradually went back to normal (Figure 4c).

#### 3.2.3. Urinalysis

##### Na^+^ and K^+^ Content in Urine

The levels of Na^+^ and K^+^ in mouse urine are shown in Table 3. Two ion concentrations in the high-dose group were much lower than those of other treated groups (*p* = 0.000), while the urine Na^+^ and K^+^ levels in the medium- and low-dose groups stayed at similar levels to those of the control. Fourteen days after suspension of treatment (day 42), the urine levels of Na^+^ and K^+^ in the high-dose group returned to normal and no longer showed any statistical difference with other groups and the control.

##### Components Detected in Urine

Detection using semiquantitative colorimetric reagent strips for all tested groups demonstrated that the main items in urine, including leukocytes, occult blood, glucose, urobilinogen, hemoglobin, albumin, microalbumin, ketone, ascorbic acid, and nitrite were all negative. Urine creatinine, uric calcium, and pH levels in the toxin treated groups were also within normal ranges.

##### Urine Specific Gravity

The results in Table 4 show that urine specific gravity in all treated groups varied little within the first week, but dropped down in the high-dose group at 2 to 3 weeks, significantly different from the other groups (*p* = 0.000). Meanwhile, the urine specific gravity in the medium- and low-dose groups remained stable, showing no statistical difference with the control. After suspension of the toxin, the urine specific gravity in the high-dose group bounced back and did not show any statistical difference between other treated groups and the control group in the following week.

#### 3.2.4. Serum Index Analysis

The interval sampling and examination of serum enzyme activity from tested subjects are shown in Table 6. TP, UA, and BUN did not show any significant changes in any groups during the overall experiment. CREA in the high-dose group decreased on the 28th day (*p* = 0.035) and went back to normal on days 35 and 42. In the high-dose group, ALT levels in mice were obviously higher than in other treated groups and kept going up with repeated administration. Seven days after the toxin was suspended, the ALT in the high-dose group started to decline, but was still higher than the control group (*p* = 0.000). Up to 14 days later, on day 42, the ALT in the high-dose group returned to normal, with no statistical difference compared with the control (*p* = 0.985). On the 28th day of the experiment, the ALT in the medium-dose group was found to be higher than that of the saline group (*p* = 0.000). Once the toxin was stopped, the ALT in the medium-dose group soon went back to normal. The enzyme AST in the high-dose group went up slightly over 1 to 2 weeks but without significant statistical difference compared with the physiological saline group. On day 28, AST in the high-dose group exhibited significantly higher levels than the control group (*p* = 0.000). Even after the toxin was suspended, AST in the high-dose group was still above the control level (*p* = 0.002). AST in the medium- and low-dose groups did not show any statistical differences with the control group. The enzyme ALP in mice in the high-dose group rose in the second week of the experiment, clearly above the other groups. When the exposure was stopped, ALP in the high-dose group went down but maintained a level above the control group. On days 14 and 28, TC in the high-dose group rose, showing a significant difference with the control group (*p* = 0.000 for the two days), whereas GLU decreased (*p* = 0.000 for the two days). Both TC and GLU returned to normal levels after the toxin was stopped. In addition, ALB in the high-dose group decreased on day 28 and went back to normal when the administration was suspended.

The serum levels of Na^+^ and K^+^ in various treated mouse groups did not show significant differences between each other and the control group, as shown in Table 3. The ADH in the blood of mice in the high-dose group was higher than that of the control on day 14 (*p* = 0.037) and day 28 (*p* = 0.000). When exposure to the toxin was stopped, blood ADH in the high-dose group went back to normal on day 35, and did not show statistical difference with the control any more. Throughout the experiment, there were no significantly statistical differences in ADH between the medium- and low-dose groups and the control group (Table 7).

#### 3.2.5. Visceral Coefficients

In the whole experiment, the kidney visceral coefficient in all tested mice in any group did not show significant differences. The autopsies of the subjects on days 14 and 28 showed that the liver visceral coefficient in the high-dose group increased compared to the control (*p* = 0.004, *p* = 0.000, respectively). After administration was stopped, the liver visceral coefficients returned to normal. A significant increase in liver visceral coefficient in the medium-dose group was seen on day 28, but no statistical differences were found in the low-dose group (Table 4).

#### 3.2.6. Histopathologic Alteration

**Figure 5:**
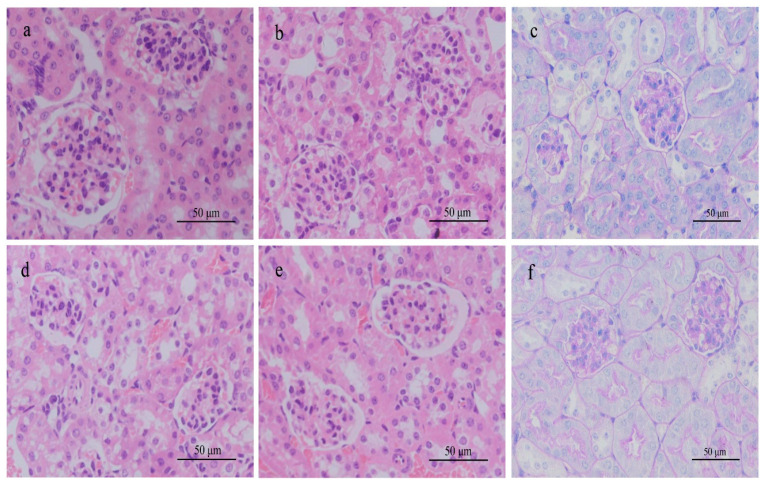
Kidneys of mice exposed to different concentrations of MC-RR for 28 days. (**a**) H & E stained histological section for 0.0 μg/kg MC-RR. (**b**) H & E stained histological section for 140.0 μg/kg MC-RR. (**d**) H & E stained histological section for 70.0 μg/kg MC-RR. (**e**) H & E stained histological section for 35.0 μg/kg MC-RR. (**c**) PAS-stained histological section for 0.0 μg/kg MC-RR. (**f**) PAS-stained histological section for 140.0 μg/kg MC-RR.

**Figure 6:**
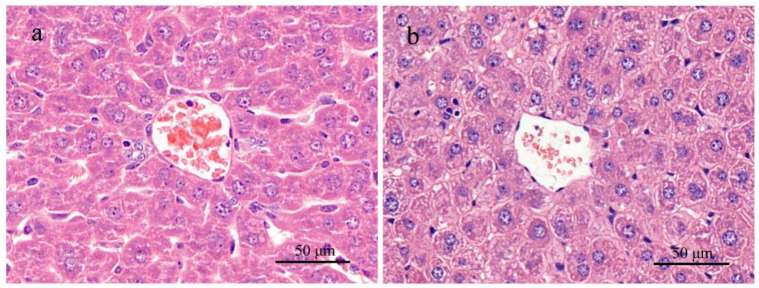
H & E stained histological sections of the livers of mice exposed to MC-RR at concentrations of 0.0 μg/kg (**a**) and 140.0 μg/kg (**b**) for 28 days.

In both HE and PAS stained sections of mouse kidneys (Figure 5), kidney microstructure was intact and clear for all tested groups. No significant lesions were detected on these kidneys in histology. In the HE stained section of mouse liver of the high-dose group, cellular swelling, ballooning changes, sinusoidal dilation, and infiltration of inflammatory cells were present on day 28 of treatment, as shown in Figure 6. For the medium- and low-dose groups, hepatic histopathologic changes were not found.

## 4. Discussion

As we know, microcystins are hepatoxins that have been confirmed in many species and reported in a number of studies [14,39,40,41,42]. Some papers have reported that the toxins also targeted other organs of tested animals [24,43,44,45]. However, more data are needed to support firm conclusions on the toxicity to other organs, such as kidneys. The kidney is a critical organ to keep water metabolism normal. Abnormal water metabolism is often identified by water intake and output, and attributed to kidney injury. However, water metabolism is a complicated process that involves several organizational systems. Kidney injury is not the only way to change water metabolism. Previous studies had various conclusions on the nephrotoxicity of MCs, but paid less attention to water metabolic changes. In the present study, the effects of MC-RR on water metabolism and the relevant nephrotoxicity in the two animals (SD rats and KM mice) were examined and compared at the same time.

In the single-dose test, the highest dose of the MC-RR was 3/4 LD_50_ with which we could better observe possible nephrotoxicity and avoid the death of the subjects. Both animals were sensitive to the given toxin and showed a series of pathological changes within 24 h. For example, rats administered 574.7 μg/kg of MC-RR (high dose) showed abnormal behaviors, such as lethargy state and anorexia. However, only rats in the high-dose group showed abnormal water metabolism, polyuria, and polydipsia. The slowed down weight gain in this group was mainly attributed to the increased urine excretion 6 h after administration started and loss of appetite. Together with the unusual urinary excretion, gross hematuria appeared in rats 12 h after administration. This means that damage might be present in the urinary system.

Further urinalysis showed a notable increase in bilirubin/urobilinogen, microalbumin, protein, and creatinine in rats given a high dose, suggesting that the toxin injured both the liver and kidney. The increased bilirubin/urobilinogen in urine came from the liver damage and the increased microalbumin, protein, and creatinine in urine pointed to kidney injury. The histologic examination strongly supported this judgment. Kidney sections stained with H&E from high-dose rats demonstrated various histopathologic changes, such as necrosis and detachment of cells, and destruction of normal kidney structures. Milutinović’s research reported damage to the kidney cortex and medulla of rats with a long-term MC-LR and MC-YR treatment in a single low dose [25]. Although the kinds of MC and the experimental method in Milutinović’s study differ from this study, kidney damage was observed after MC treatment in both. The kidney tubule is the place where water and salt reabsorption occur [46]. Damage to the kidney tubules resulted in polyuria and high Na^+^ with low K^+^ in urine in this study, especially in the high-dose group. Serious polyuria then caused the polydipsia.

In the medium-dose (287.3 μg/kg) group of rats, urine protein and occult blood were detected with histologic alteration of inflammable cell infiltration, but such toxic effect was definitely mild compared to the high-dose group. Although the symptoms and histopathologic changes were not observed in rats given the low dose (143.7 μg/kg) group, the abnormal higher concentration of Na^+^ and lower K^+^ in urine found in this group may be more sensitive indices that reflected the early or slight damage of the kidney by MC-RR. Within 24 h, from clinical symptoms to histopathology and laboratory analysis, all examination evidence proved that the rat kidneys were targeted by high, medium, and low doses of MC-RR to some extent. These results are similar to Lowe’s report, in which he used only a single dose of MC-LR to observe the nephrotoxicity [47]. Although the toxin and dosage used were different between his study and this one, the pathologic changes in rats were similar. In this study, gradient dosages were used so that a dose effect could be observed. However, no dysfunction or pathologic alteration in the mouse urinary system and kidney were observed with any toxin dosage given, whereas liver damage was prominent.

The serologic analysis showed that many key enzymatic activities reflecting the function of the kidney and liver in rats were higher or lower in the high- and medium-dose groups than the control, indicating that the related organs were damaged and the enzymes were released into the blood. For mice, nonspecific enzymes, such as ALT, AST, ALP, and LDH were abnormally in the blood serum in the high-dose group, suggested certain organ damage. Combined with the increased TC and decreased GLU in the high-dose group, the liver injury was mostly confirmed. The histopathologic liver changes in the high-dose group also confirmed liver damage. However, no kidney-related specificity index was found to have an abnormal level in mice. The Na^+^ in blood of the high-dose rat group was lower and the K^+^ level was higher than the control. Such a trend echoed their levels in urine, but this urine change could be observed in all rat groups, not exclusively limited to the high dose-group. The Na^+^ and K^+^ changes in blood lagged behind those in urine. The large amount of water and Na^+^ lost from the urine in the high-dose group was the main reason why the changes of Na^+^ and K^+^ levels in blood in this group were more obvious than in other groups.

In terms of the pathogenic symptoms and possible mechanisms, the intervention measure, DXM, was used to examine the offset effects on pathogenic phenomena in the present investigation. When different doses of DXM (50.0, 10.0, and 2.0 mg/kg) combined with 574.7 μg/kg of MC-RR was administered to rats, the overall pathologic symptoms and histopathologic lesions were improved, showing a good dose-response relationship. These results suggest that DXM, a universal anti-inflammatory drug, could counter the acute damage caused by MC-RR. From another angle, the polydipsia polyuria relief occuring under DXM administration also suggests that the polydipsia polyuria observed in rats was mainly caused by kidney injury. There was corroboration between this part of the results and Nobre’s study, which reported that the DXM could block the increased glomerular filtration rate and urinary flow caused by MC-LR in the isolated perfused rat kidney [19]. The differences in the kidney effect between rats and mice under single dose MC-RR treatment were obvious and in sharp contrast. Though the high dose for the two animals was 3/4 LD_50_, the nephrotoxocity was explicit in rats but was not reflected in mice, implying that SD rat kidney is more sensitive than KM mouse kidney to MC-RR exposure. The reasons for this difference in the two animals have not been fully clarified. Previous studies suggested that the MCs enter the cells or tissues through organic anion transporting polypeptides (Oatps), which include several members [43,48,49,50]. The kinds of Oatps distributed in rat and mouse kidneys are not exactly the same [51,52,53,54,55]. Maybe the types and content of Oatps in the two animals’ kidneys are part of the reasons for the different response to MC-RR.

Since abnormal water metabolism in rats was found to be mainly caused by the kidney damage in the single dose toxicity experiment, the continuous exposure test was performed on the mice in the present study. In the continuous exposure experiment, the weight gain of mice in receiving a high dose (140.0 μg/kg) started to slow down on day 9 and water intake increased on the same day, because the urine output was increased on day 8 ahead of the weight loss and increased water intake. The three symptoms became worse with repeated administration until the toxin was stopped on day 29, indicating that there was a problem in water metabolism. It seems that the urinary system and kidney function should be responsible for such pathologic phenomena, since the symptoms were quite like rat intoxication. However, further urine and serum analysis did not support this assumption, because the indices reflecting kidney function in urine and blood were almost all within normal ranges, except specific gravity and levels of Na^+^ and K^+^ in mouse urine; in the urine of mice in the high-dose group, they were significant lower than the control simply due to the large volume of urine diluting them.

A different scenario could be seen with rats in this experiment, in that their levels of Na^+^ and K^+^ were quite opposite to their abnormal level, caused by kidney damage. The histological examination of mice given a high dose showed that the structure of the kidney under the microscope was intact and well arranged just like the control group. Therefore, the evidence to associate kidney damage with water metabolic dysfunction was not yet found in mice, even though the toxicity test extended to the 42nd day. Hooser and Qin’s research may support this part of the results, that no significant kidney injury was found in mice after MC administration in the acute experiment [56] or long-term exposure [26], although these studies used MC-LR and paid no attention to the related water metabolism. The dysfunction of water metabolism in rats and mice treated with MC-RR was compared for the first time in the present study.

We expected that the toxin would affect the livers of mice in the high-dose group when samples were collected for the first time on day 7, showing increasing serum ALT. Other serum indices (AST, ALP, and TC) started to increase around day 14 and returned to normal or were relieved one or two weeks after administration was stopped on day 29. This trend was also shown in histologic liver changes. When the toxin was given, the liver sections from mice in the high-dose group showed various lesions; however, when the toxin was stopped, the histologic liver changes did not show evident lesions any more. The continuous exposure experiment demonstrated that the liver damage in mice by high-dose toxin was reversible. As long as the mice were not exposed to the MC-RR, all the abnormal values went back to normal levels.

The polydipsia polyuria of the mice in the high-dose group showed the same change trend as the liver damage, suggesting that the abnormal water metabolism was synchronous with the MC-RR exposure and more related to the abnormal liver system rather than the kidney. The changes of ADH in the high-dose group were mainly considered as feedback regulation. Water metabolism involves several organs and systems, not only the kidney [57]. When water metabolism is found to be abnormal, it implies injury to the relative systems. In Zhong’s study, water metabolism dysfunction induced by MC-RR in mice was thought to be caused by liver damage [58]. Zhao’s research reported similar polydipsia induced by MC-LR and attributed the polydipsia to the thyroid dysfunction, but they did not pay attention to the urine volume [59]. Those two reports suggested that the polydipsia was caused by dysfunction of the thyroid or liver, not the kidney. On the other hand, Yi evaluated the influence of chronic exposure to MC-LR on mouse kidney, and found kidney injury after three months [30]. Thus, although the kidney damage indices were not found in our study, the possibility of dysfunction originating from the kidney cannot be easily excluded. The real mechanism of polydipsia polyuria that occurred in mice intoxicated by MC-RR is not yet clear. The normal level K^+^ in blood indicated that the polydipsia polyuria was not a result of hypokalemia and aldosteronism. The low blood glucose levels and increased ADH in mice also exclude diabetes and diabetes insipidus, which are common causes of polydipsia polyuria [60,61]. The outcomes from the present study offer two possible causes: dysfunction originating in the kidney without parenchymal damage, and liver-related metabolic dysfunction. More extensive and detailed research is needed to find the mechanism.

## 5. Conclusions

In the present study, possible abnormal water metabolism induced by microcystin-RR in SD rats and KM mice was explored and compared. The results show that rats administered MC-RR intraperitoneally at a dose of 574.7 μg/kg (3/4 LD_50_ dose) exhibited polydipsia and polyuria with gross hematuria, urinary microalbumin and protein, and histopathologic kidney changes within 24 h. On the contrary, mice administered MC-RR at a dose of 210.0 μg/kg (3/4 LD_50_ dose) did not display obvious abnormalities in drinking and urination in a single-dose toxicity test. Kidney injury was not demonstrated in mice with negative outcomes by macro- and micro-examination. DXM could neutralize the nephrotoxicity caused by the toxin and effectively alleviate the pathologic kidney symptoms and signs in rats. A continuous exposure experiment was performed on mice for 42 days. Mice receiving 140.0 μg/kg MC-RR daily showed polydipsia polyuria on day 9 that became worse with time. Nevertheless, the water metabolic dysfunction was reversible when administration of the toxin was stopped. The overall examination of the mice, including postmortem, serum enzymes and histopathology, did not show an association of the symptoms with the kidney. The changing patterns of polydipsia polyuria were consistent with liver damage under MC-RR treatment, indicating that some other MC-triggered metabolic pathways or liver-related metabolic route might play a role. In a word, polydipsia and polyuria may be induced by MC-RR in SD rats and KM mice with different causes or metabolic pathways. The SD rat kidney is more sensitive to MC-RR compared with the KM mice kidney. High-dose MC-RR can cause water metabolic dysfunction in rats by damaging the kidney, but cannot affect the drinking volume and urine volume of mice in single-dose exposure. Continuous exposure of high-dose MC-RR can induce abnormal water metabolism in mice without significant kidney injury.

## Figures and Tables

**Table 1:** Water intake, urine output and weight gain of Sprague–Dawley (SD) rats and KunMing (KM) mice treated with microcystin-RR (MC-RR) in single-dose experiment (mean ± SD, *n* = 6).

	Group	Water Intake (10^−1^ mL/g/24 h)		Urine Output (10^−^^2^ mL/g/24 h)		Weight Gain (g/24 h)	
		Before Test	After Administration	Before Test	After Administration	Before Test	After Administration
SD Rat	Saline control	1.50 ± 0.047	1.48 ± 0.076	4.43 ± 0.314	4.45 ± 0.396	9.71 ± 3.155	10.78 ± 3.023
	143.7 μg/kg MC-RR	1.44 ± 0.050	1.53 ± 0.100	4.03 ± 0.414	4.22 ± 0.740	9.12 ± 1.477	8.69 ± 1.441
	287.3 μg/kg MC-RR	1.46 ± 0.032	1.44 ± 0.046	4.18 ± 0.411	4.35 ± 0.665	8.31 ± 3.262	9.28 ± 2.124
	574.7 μg/kg MC-RR	1.55 ± 0.083	1.84 ± 0.112 *^α^	4.35 ± 0.639	16.79 ± 1.069 *^α^	9.94 ± 1.394	3.53 ± 2.358 *^α^
KM Mouse	Saline control	2.83 ± 0.032	2.72 ± 0.103	2.56 ± 0.116	2.62 ± 0.061	2.08 ± 0.479	2.63 ± 0.686
	52.5 μg/kg MC-RR	2.88 ± 0.075	2.83 ± 0.067	2.71 ± 0.0714	2.61 ± 0.095	2.45 ± 0.589	2.62 ± 0.534
	105.0 μg/kg MC-RR	2.78 ± 0.112	2.79 ± 0.054	2.74 ± 0.090	2.75 ± 0.139	2.18 ± 0.475	2.25 ± 0.413
	210.0 μg/kg MC-RR	2.85 ± 0.069	2.83 ± 0.043	2.63 ± 0.083	2.68 ± 0.018	2.40 ± 0.379	2.05 ± 0.446
SD Rat	Saline control	1.48 ± 0.024	1.50 ± 0.023	4.38 ± 0.213	4.42 ± 0.287	10.28 ± 2.536	9.23 ± 1.807
	0.0 mg/kg DXM + MC-RR 574.7 μg/kg	1.49 ± 0.053	1.86 ± 0.044 * ^α^	4.47 ± 0.509	17.72 ± 0.659 * ^α^	9.12 ± 2.565	3.48 ± 0.916 * ^α^
	2.0 mg/kg DXM + MC-RR 574.7 μg/kg	1.50 ± 0.068	1.58 ± 0.043 * ^#^	4.16 ± 0.340	15.7 ± 0.871 * ^α^	9.84 ± 1.564	5.06 ± 1.679 * ^α^
	10.0 mg/kg DXM + MC-RR 574.7 μg/kg	1.487 ± 0.080	1.51 ± 0.040 ^#^	3.94 ± 0.258	8.98 ± 0.793 ^#^^α^	9.49 ± 1.771	7.13 ± 1.330 * ^# α^
	50.0 mg/kg DXM + MC-RR 574.7 μg/kg	1.50 ± 0.058	1.50 ± 0.053 ^#^	4.01 ± 0.395	4.24 ± 0.876 ^#^	9.85 ± 1.672	9.53 ± 2.192 ^#^

* Significantly different from the saline control group (0.0 μg/kg MC-RR), *p* < 0.05; # Significantly different from the 0.0 mg/kg DXM + MC-RR 574.7 μg/kg group, *p* < 0.05; α Significantly different from the self-control (before test), *p* < 0.05.

**Table 2:** Urinalysis of SD rats and KM mice treated with MC-RR in single dose experiment detected by the semiquantitative colorimetric reagent strip.

	Group	Leukocyte (Leu/μL)	Urobilinogen (μmol/L)	Micro-albumin (mg/L)	Protein (g/L)	Bilirubin (mmol/L)	Glucose (mmol/L)	Ascorbic Acid (mmol/L)	Ketone (mmol/L)	Nitrite *	Creatinine (mmol/L)	pH	Occult Blood (Ery/μL)	Calcium (mmol/L)
SD Rat	Saline control	<15	≤3.5	10−30	<0.15	<17.0	<2.8	<0.56	<0.5	Negative	≤0.9	7.5–8.5	<10	5.0–10.0
	143.7 μg/kg MC-RR	<15	≤3.5	10−30	<0.15	<17.0	<2.8	<0.56	<0.5	Negative	≤0.9	7.5–8.0	<10	5.0–10.0
	287.3 μg/kg MC-RR	≤15	≤3.5	10−80	0.15–0.30	<17.0	<2.8	<0.56	<0.5	Negative	≤0.9	7.0–8.5	10–25	5.0–10.0
	574.7 μg/kg MC-RR	70–125	70–140	30–150	0.15–1.00	<17.0	<2.8	<0.56	0.5–1.5	Negative	0.9–4.4	6.0–7.5	80–200	1.0–2.5
KM Mouse	Saline control	<15	≤3.5	≤10	<0.15	<17.0	<2.8	<0.56	<0.5	Negative	≤0.9	7.0–8.5	<10	5.0–10.0
	52.5 μg/kg MC-RR	<15	≤3.5	≤10	<0.15	<17.0	<2.8	<0.56	<0.5	Negative	≤0.9	7.5–8.5	<10	5.0–10.0
	105.0 μg/kg MC-RR	<15	≤3.5	≤10	<0.15	<17.0	<2.8	<0.56	<0.5	Negative	≤0.9	7.5–8.5	<10	5.0–10.0
	210.0 μg/kg MC-RR	<15	≤3.5	≤10	<0.15	<17.0	<2.8	<0.56	<0.5	Negative	≤0.9	7.0–8.0	<10	2.5–10.0
SD Rat	Saline control	<15	≤3.5	10–30	<0.15	<17.0	<2.8	<0.56	<0.5	Negative	≤0.9	7.5–8.5	<10	5.0–10.0
	0.0 mg/kg DXM + MC-RR 574.7 μg/kg	70–125	140–200	80–150	0.15–1.00	≤17.0	<2.8	<0.56	<0.5	Negative	0.9–17.7	6.0–7.0	80–200	1.0–2.5
	2.0 mg/kg DXM + MC-RR 574.7 μg/kg	15–70	140–200	80–150	0.15–1.00	≤17.0	<2.8	<0.56	< 0.5	Negative	0.9–17.7	5.0–7.0	80–200	1.0–5.0
	10.0 mg/kg DXM + MC-RR 574.7 μg/kg	15–70	17–200	80–150	0.15–1.00	<17.0	<2.8	0.00–1.14	<0.5	Negative	0.9–8.8	6.0–7.5	10–25	2.5–5.0
	50.0 mg/kg DXM + MC-RR 574.7 μg/kg	<15	≤3.5	80–150	0.15–0.30	<17.0	<2.8	0.56–1.14	<0.5	Negative	≤0.9	6.5–7.5	≤10	2.5–5.0

* Qualitative test for this index with the result described by “Negative” or “Positive”.

**Table 3:** Concentration of Na+ and K+ in urine and serum for SD rat and KM mouse (mean ± SD).

	Animal	Time	MC-RR Dosage (μg/kg)	Na^+^ in Urine (mg/L)	K^+^ in Urine (mg/L)	Na^+^ in Serum (mg/L)	K^+^ in Serum (mg/L)
Single dose experiment (*n* = 6)	SD Rat	24 h	0.0	3368.6 ± 202.61	10031.2 ± 469.04	3723.0 ± 158.36	278.9 ± 34.50
			143.7	4169.3 ± 290.28 *	9193.0 ± 371.53 *	3670.9 ± 115.45	277.8 ± 33.18
			287.3	6183.0 ± 234.86 *	8363.7 ± 450.92 *	3443.9 ± 183.78 *	283.1 ± 32.33
			574.7	7812.5 ± 436.24 *	3443.8 ± 221.59 *	3165.7 ± 192.05 *	362.5 ± 30.04 *
	KM Mouse	24 h	0.0	3041.4 ± 302.81	7717.4 ± 89.71	3303.3 ± 241.35	281.3 ± 12.17
			52.5	3256.4 ± 244.92	7919.8 ± 262.83	3077.7 ± 131.42	276.2 ± 18.32
			105.0	2974.5 ± 219.12	7826.1 ± 283.90	3148.8 ± 80.68	265.6 ± 11.00
			210.0	3119.5 ± 226.07	7985.3 ± 298.80	3208.9 ± 139.36	297.7 ± 13.33
Continuous exposure experiment (*n* = 7)	KM Mouse	28th day	0.0	3174.8 ± 387.07	7918.2 ± 242.43	3107.1 ± 93.03	265.4 ± 20.76
			35.0	3033.6 ± 243.71	7916.4 ± 232.30	3136.7 ± 98.92	272.5 ± 32.68
			70.0	2885.2 ± 364.97	7797.5 ± 272.12	3210.2 ± 82.94	260.0 ± 25.24
			140.0	989.6 ± 244.02 *	1336.4 ± 101.25 *	3171.9 ± 41.68	263.8 ± 19.09
		42nd day	0.0	2979.4 ± 244.21	7874.9 ± 179.49	3182.4 ± 153.93	273.8 ± 23.13
			35.0	3012.2 ± 237.52	8021.0 ± 219.97	3308.8 ± 110.69	276.6 ± 14.21
			70.0	2989.6 ± 219.58	7966.3 ± 210.53	3284.4 ± 111.91	280.6 ± 32.61
			140.0	2855.2 ± 250.43	7807.9 ± 292.55	3175.3 ± 95.78	259.9 ± 15.97

* Significantly different from the saline control group (0.0 μg/kg MC-RR), *p* < 0.05.

**Table 4:** Organ coefficient and urine specific gravity of SD rats and KM mice (mean ± SD).

	Animal	Time	MC-RR Dosage (μg/kg)	Urine Specific Gravity	Organ Coefficient (% of Body Weight)	
					Kidney	Liver
Single dose experiment (*n* = 6)	SD Rat	24 h	0.0	1.032 ± 0.0123	0.911 ± 0.078	4.77 ± 0.081
			143.7	1.041 ± 0.0182	0.958 ± 0.053	4.68 ± 0.231
			287.3	1.031 ± 0.0138	0.977 ± 0.124	4.97 ± 0.656
			574.7	1.020 ± 0.0058	1.123 ± 0.207	5.13 ± 0.275
	KM Mouse	24 h	0.0	1.053 ± 0.0024	1.38 ± 0.102	5.60 ± 0.400
			52.5	1.054 ± 0.0058	1.33 ± 0.141	5.72 ± 0.344
			105.0	1.056 ± 0.0050	1.39 ± 0.055	5.43 ± 0.457
			210.0	1.052 ± 0.0046	1.31 ± 0.153	5.60 ± 0.400
Continuous exposure experiment (*n* = 7)	KM Mouse	7th day	0.0	1.053 ± 0.0044	1.29 ± 0.132	5.64 ± 0. 410
			35.0	1.055 ± 0.0053	1.18 ± 0.064	5.69 ± 0.259
			70.0	1.054 ± 0.0060	1.24 ± 0.056	5.95 ± 0.315
			140.0	1.054 ± 0.0064	1.31 ± 0. 101	5.89 ± 0. 260
		14th day	0.0	1.054 ± 0.0060	1.26 ± 0037	4.96 ± 0.182
			35.0	1.052 ± 0.0056	1.39 ± 0.167	5.39 ± 0.570
			70.0	1.051 ± 0.0035	1.37 ± 0.221	5.22 ± 0.501
			140.0	1.018 ± 0.0077 *	1.32 ± 0.104	5.86 ± 0.522 *
		28th day	0.0	1.055 ± 0.0054	1.29 ± 0.092	4.77 ± 0.169
			35.0	1.054 ± 0.0057	1.29 ± 0.141	4.92 ± 0.230
			70.0	1.053 ± 0.0072	1.39 ± 0.129	5.69 ± 0.446 *
			140.0	1.015 ± 0.0076 *	1.25 ± 0.101	5.66 ± 0.305 *
		42nd day	0.0	1.054 ± 0.0070	1.40 ± 0.180	5.04 ± 0.265
			35.0	1.055 ± 0.0053	1.26 ± 0.109	5.27 ± 0.435
			70.0	1.053 ± 0.0051	1.36 ± 0.092	5.20 ± 0.485
			140.0	1.056 ± 0.0078	1.25 ± 0.093	5.01 ± 0.571

* Significantly different from the saline control group (0.0 μg/kg MC-RR), *p* < 0.01.

**Table 5:** Serum biochemical analysis of SD rats and KM mice treated with MC-RR in single dose experiment (mean ± SD, *n* = 6).

	Group	Creatinine (mmol/L)	Uric Acid (mmol/L)	Urea Nitrogen (mmol/L)	Alanine Aminotransferase (IU/L)	Glutamic Oxaloacetic transaminase (IU/L)	Alkaline Phosphatase (IU/L)	Lactic Dehydrogenase (IU/L)	Albumin (g/L)	Glucose (mmol/L)	Total Cholesterol (mmol/L)	Total Protein (g/L)
SD Rat	Saline control	35.43 ± 6.705	133.72 ± 25.46	2.99 ± 0.455	44.15 ± 8.943	136.37 ± 29.228	412.91 ± 31.575	376.16 ± 37.784	32.45 ± 3.256	7.79 ± 0.374	1.70 ± 0.374	51.93 ± 6.049
	143.7 μg/kg MC-RR	28.75 ± 4.127	105.13 ± 11.647	3.73 ± 0.638	55.10 ± 7.539	204.41 ± 47.776	436.81 ± 57.679	277.99 ± 48.723	33.27 ± 2.121	7.858 ± 0.997	1.61 ± 0.274	52.35 ± 5.329
	287.3 μg/kg MC-RR	38.07 ± 9.047	123.20 ± 19.398	4.55 ± 0.714	87.43 ± 10.936 **	341.12 ± 61.419 **	504.29 ± 75.969	298.91 ± 65.919	33.05 ± 0.695	7.416 ± 1.077	1.90 ± 0.139	52.53 ± 2.109
	574.7 μg/kg MC-RR	48.13 ± 11.566	77.23 ± 12.371 **	4.61 ± 2.091	976.07 ± 231.512 **	1215.17 ± 130.027**	842.45 ± 75.342 **	1318.13 ± 325.223 **	33.07 ± 2.778	6.844 ± 0.719	1.82 ± 0.264	57.90 ± 11.013
KM Mouse	Saline control	27.13 ± 2.099	153.68 ± 5.070	3.73 ± 0.166	34.90 ± 2.672	130.21 ± 11.895	189.08 ± 9.653	479.06 ± 9.112	34.30 ± 1.396	6.31 ± 0.449	2.59 ± 0.246	60.65 ± 1.379
	52.5 μg/kg MC-RR	26.71 ± 2.0645	150.92 ± 4.540	3.73 ± 0.246	37.66 ± 2.678	140.66 ± 10.483	187.53 ± 7.363	499.56 ± 21.265	34.23 ± 0.569	6.52 ± 0.326	2.54 ± 0.141	59.38 ± 4.845
	105.0 μg/kg MC-RR	27.17 ± 3.602	153.53 ± 4.950	3.84 ± 0.405	40.59 ± 4.171 *	134.65 ± 10.288	184.80 ± 19.889	510.73 ± 44.978	33.57 ± 1.123	6.07 ± 0.468	2.68 ± 0.193	59.53 ± 1.734
	210.0 μg/kg MC-RR	24.73 ± 2.562	144.18 ± 14.960	4.09 ± 0.587	63.83 ± 4.134 **	185.86 ± 8.770**	272.07 ± 7.567 **	552.91 ± 21.362 **	34.98 ± 1.961	6.40 ± 0.699	2.67 ± 0.299	58.95 ± 2.688
SD Rat	Saline control	ND	ND	ND	47.47 ± 7.847	144.84 ± 23.050	408.58 ± 53.478	317.15 ± 49.304	ND	ND	ND	ND
	0.0 mg/kg DXM + MC-RR 574.7 μg/kg	ND	ND	ND	1001.43 ± 2.672 **	1223.33 ± 200.147 **	866.39 ± 86.98 **	1268.86 ± 285.334 **	ND	ND	ND	ND
	2.0 mg/kg DXM + MC-RR 574.7 μg/kg	ND	ND	ND	625.16 ± 26.054 ** ^#^	1354.57 ± 51.446**^#^	697.09 ± 14.698 ** ^#^	1002.08 ± 40.163 **	ND	ND	ND	ND
	10.0 mg/kg DXM + MC-RR 574.7 μg/kg	ND	ND	ND	93.41 ± 21.091 * ^##^	361.38 ± 18.384**^##^	564.82 ± 19.894** ^##^	583.84 ± 19.245 ** ^##^	ND	ND	ND	ND
	50.0 mg/kg DXM + MC-RR 574.7 μg/kg	ND	ND	ND	70.17 ± 13.115 * ^##^	190.78 ± 15.600 * ^##^	486.85 ± 13.374 ^##^	514.94 ± 31.356 ** ^##^	ND	ND	ND	ND

* Significantly different from the saline control group (0.0 μg/kg MC-RR), *p* < 0.05; ** Significantly different from the saline control group (0.0 μg/kg MC-RR), *p* < 0.01; # Significantly different from the 0.0 mg/kg DXM + 574.7 μg/kg MC-RR group, *p* < 0.05; ## Significantly different from the 0.0 mg/kg DXM + 574.7 μg/kg MC-RR group, *p* < 0.01; ND: No detection.

**Table 6:** Serum biochemical analysis of KM mice treated with MC-RR in continuous exposure experiment (mean ± SD, *n* = 7).

	MC-RR Dosage (μg/kg)	Albumin (g/L)	Alanine Aminotransferase (IU/L)	Alkaline Phosphatase (IU/L)	Glutamic Oxaloacetic Transaminase (IU/L)	Urea Nitrogen (mmol/L)	Creatinine (mmol/L)	Glucose (mmol/L)	Total Cholesterol (mmol/L)	Total Protein (g/L)	Uric Acid (mmol/L)
7th day	0.0	30.23 ± 0.568	42.30 ± 3.255	54.93 ± 13.380	141.41 ± 14.025	4.09 ± 0.317	71.44 ± 4.106	9.08 ± 0.630	2.05 ± 0.288	62.26 ± 1.215	119.96 ± 20.052
	35.0	31.01 ± 1.111	55.21 ± 13.65	45.57 ± 10.886	144.63 ± 20.986	4.10 ± 0.231	70.66 ± 4.154	9.12 ± 0.741	2.12 ± 0.292	62.04 ± 1.868	110.39 ± 21.005
	70.0	29.91 ± 1.027	55.21 ± 13.655	67.07 ± 22.606	147.61 ± 21.026	4.06 ± 0.270	72.07 ± 3.214	8.63 ± 0.514	2.14 ± 0.252	61.23 ± 0.976	108.70 ± 20.921
	140.0	30.56 ± 0.881	98.48 ± 27.081 *	92.51 ± 25.885 *	188.69 ± 58.831	3.96 ± 0.281	69.93 ± 4.889	8.14 ± 0.986	2.23 ± 0.358	60.49 ± 1.503	103.54 ± 22.823
14th day	0.0	30.16 ± 0.789	44.14 ± 10.127	60.19 ± 8.999	154.03 ± 16.749	4.00 ± 0.263	70.46 ± 3.179	9.24 ± 0.38	1.99 ± 0.268	60.78 ± 1.831	110.99 ± 15.422
	35.0	29.53 ± 0.976	44.17 ± 6.766	56.00 ± 14.439	156.71 ± 18.609	4.02 ± 0.373	70.29 ± 2.764	9.16 ± 1.011	1.99 ± 0.253	62.00 ± 1.547	109.91 ± 23.107
	70.0	30.26 ± 1.121	50.24 ± 6.710	65.31 ± 7.371	146.17 ± 26.161	3.90 ± 0.283	69.14 ± 3.580	7.55 ± 0.296 *	2.14 ± 0.297	60.91 ± 1.720	112.68 ± 15.416
	140.0	29.97 ± 0.450	137.14 ± 22.760 *	96.77 ± 9.122 *	193.214 ± 44.578	4.00 ± 0.246	69.94 ± 2.407	6.55 ± 0.372 *	3.12 ± 0.321 *	61.14 ± 1.367	114.94 ± 19.323
28th day	0.0	30.09 ± 0.778	42.53 ± 7.489	64.21 ± 6.564	145.03 ± 13.778	4.08 ± 0.206	71.20 ± 2.258	10.07 ± 0.562	1.99 ± 0.228	61.34 ± 1.852	110.70 ± 18.762
	35.0	29.91 ± 0.919	43.16 ± 6.312	61.74 ± 10.527	143.27 ± 16.635	4.14 ± 0.228	69.14 ± 3.723	9.27 ± 0.853	2.03 ± 0.266	60.14 ± 2.052	109.77 ± 18.858
	70.0	28.87 ± 1.411	63.80 ± 5.525 *	64.11 ± 7.049	157.71 ± 12.444	4.05 ± 0.381	70.93 ± 3.119	7.78 ± 0.432 *	2.06 ± 0.252	61.39 ± 1.631	115.69 ± 19.889
	140.0	26.46 ± 0.866 *	148.26 ± 9.466 *	133.97 ± 22.060 *	214.042 ± 24.292 *	3.97 ± 0.308	66.87 ± 3.458 *	6.04 ± 0.465 *	2.84 ± 0.462 *	62.10 ± 2.036	112.54 ± 18.587
35th day	0.0	29.60 ± 0.616	39.60 ± 6.119	63.56 ± 11.527	147.79 ± 7.247	3.98 ± 0.309	71.31 ± 3.050	9.43 ± 1.019	2.09 ± 0.252	61.60 ± 1.910	114.50 ± 15.150
	35.0	29.76 ± 0.768	38.64 ± 6.274	64.36 ± 9.727	142.89 ± 7.623	3.89 ± 0.269	70.66 ± 2.667	9.28 ± 0.575	2.07 ± 0.167	60.71 ± 2.023	117.93 ± 18.973
	70.0	30.00 ± 0.929	41.17 ± 4.285	66.14 ± 6.432	153.57 ± 10.916	4.03 ± 0.258	72.13 ± 4.162	8.69 ± 0.655	2.13 ± 0.269	62.14 ± 1.682	113.73 ± 11.466
	140.0	29.64 ± 0.907	70.00 ± 13.075 *	111.286 ± 6.048 *	186.53 ± 9.739 *	4.08 ± 0.315	71.93 ± 3.949	8.96 ± 0.460	2.32 ± 0.427	61.73 ± 1.227	119.43 ± 18.655
42nd day	0.0	30.08 ± 0.667	37.96 ± 4.184	65.34 ± 8.383	153.06 ± 17.171	4.02 ± 0.295	71.73 ± 3.899	9.42 ± 0.524	2.19 ± 0.341	62.26 ± 2.811	119.26 ± 16.825
	35.0	31.07 ± 0.637	35.49 ± 4.416	62.50 ± 9.637	160.93 ± 11.012	3.97 ± 0.331	71.40 ± 4.477	9.28 ± 0.575	2.22 ± 0.312	61.80 ± 2.131	116.23 ± 13.663
	70.0	30.46 ± 0.688	34.07 ± 4.320	67.63 ± 8.176	169.69 ± 13.620	4.11 ± 0.304	72.24 ± 3.644	8.69 ± 0.655	2.15 ± 0.317	62.34 ± 2.609	113.24 ± 12.241
	140.0	29.51 ± 1.104	38.79 ± 8.107	91.24 ± 14.434 *	183.40 ± 16.591 *	4.01 ± 0.333	70.00 ± 2.831	8.96 ± 0.460	2.28 ± 0.371	60.60 ± 2.011	117.29 ± 15.093

* Significantly different from the saline control group (0.0 μg/kg MC-RR), *p* < 0.05.

**Table 7:** Antidiuretic hormone (ADH) levels of KM mice in continuous exposure experiment (mean ± SD, *n* = 7).

MC-RR Dosage (μg/kg)	7th Day	14th Day	28th Day	35th Day	42nd Day
0.0	203.60 ± 25.491	208.69 ± 33.740	198.60 ± 11.229	196.99 ± 26.105	204.94 ± 35.174
35.0	198.51 ± 13.311	192.44 ± 21.701	189.58 ± 37.184	188.87 ± 24.173	205.29 ± 28.520
70.0	207.17 ± 37.415	218.27 ± 26.093	218.24 ± 22.832	200.12 ± 19.228	196.82 ± 23.195
140.0	198.96 ± 26.346	228.15 ± 24.805 *	267.79 ± 20.126 **	189.31 ± 17.105	203.78 ± 24.784

* Significantly different from the saline control group (0.0 μg/kg MC-RR), *p* < 0.05; ** Significantly different from the saline control group (0.0 μg/kg MC-RR), *p* < 0.01.

## Supplementary Materials

The following are available online at https://www.mdpi.com/1660-4601/18/4/1900/s1, Figure S1: Chromatograms of MC-RR standard (a) and the purified MC- RR (b and c).

## References

[1] Chen L., Giesy J.P., Adamovsky O., Svirčev Z., Meriluoto J., Codd G.A., Mijovic B., Shi T., Tuo X., Li S.-C. (2021). Challenges of using blooms of Microcystis spp. in animal feeds: A comprehensive review of nutritional, toxicological and microbial health evaluation. Sci. Total Environ..

[2] Bouaïcha N., Miles C.O., Beach D.G., Labidi Z., Djabri A., Benayache N.Y., Nguyen-Quang T. (2019). Structural diversity, characterization and toxicology of microcystins. Toxins.

[3] Nasri A.-B., Bouaïcha N., Fastner J. (2004). First report of a microcystin-containing bloom of the Cyanobacteria *Microcystis* spp. in Lake Oubeira, Eastern Algeria. Arch. Environ. Contam. Toxicol..

[4] Mankiewicz J., Komárková J., Izydorczyk K., Jurczak T., Tarczyńska M., Zalewski M. (2005). Hepatotoxic cyanobacterial blooms in the lakes of northern Poland. Environ. Toxicol..

[5] Znachor P., Jurczak T., Komárková J., Jezberová J., Mankiewicz J., Kaštovská K., Zapomělová E. (2006). Summer changes in cyanobacterial bloom composition and microcystin concentration in eutrophic Czech reservoirs. Environ. Toxicol..

[6] Su X., Xue Q., Stinman A.D., Zhao Y., Xie L. (2015). Spatiotemporal dynamics of microcystin variants and relationships with environmental parameters in Lake Taihu, China. Toxins.

[7] Nishiwaki-Matsushima R., Ohta T., Nishiwaki S., Suganuma M., Kohyama K., Ishikawa T., Carmichael W.W., Fujiki H. (1992). Liver tumor promotion by the cyanobacterial cyclic peptide toxin microcystin-LR. J. Cancer Res. Clin. Oncol..

[8] Ito E., Kondo F., Terao K., Harada K.-I. (1997). Neoplastic nodular formation in mouse liver induced by repeated intraperitoneal injections of microcystin-LR. Toxicon.

[9] Yoshida T., Makita Y., Nagata S., Tsutsumi T., Yoshida F., Sekijima M., Tamura S., Ueno Y. (1997). Acute oral toxicity of microcystin-LR, a cyanobacterial hepatotoxin, in mice. Nat. Toxins.

[10] Jochimsen E.M., Carmichael W.W., An J., Cardo D.M., Cookson S.T., Holmes C.E.M., Antunes M.B., Filho D.A.D.M., Lyra T.M., Barreto V.S.T. (1998). Liver failure and death after exposure to microcystins at a hemodialysis center in Brazil. N. Engl. J. Med..

[11] Herfindal L., Selheim F. (2006). Microcystin produces disparate effects on liver cells in a dose dependent manner. Mini-Rev. Med Chem..

[12] Ohta T., Nishiwaki R., Yatsunami J., Komori A., Suganuma M., Fujiki H. (1992). Hyperphosphorylation of cytokeratins 8 and 18 by microcystin-LR, a new liver tumor promoter, in primary cultured rat hepatocytes. Carcinogenesis.

[13] Frangez R., Kosec M., Sedmak B., Beravs K., Demsar F., Juntes P., Pogacnik M., Suput D. (2000). Subchronic liver injuries caused by microcystins. Pflugers Arch. Eur. J. Phy..

[14] Zhang Z., Zhang X.-X., Wu B., Yin J., Yu Y., Yang L. (2016). Comprehensive insights into microcystin-LR effects on hepatic lipid metabolism using cross-omics technologies. J. Hazard. Mater..

[15] Rai A.K., Chaturvedi R., Kumar A. (2018). Proteomic evidences for microcystin-RR-induced toxicological alterations in mice liver. Sci. Rep..

[16] Kubickova B., Babica P., Hilscherová K., Šindlerová L. (2019). Effects of cyanobacterial toxins on the human gastrointestinal tract and the mucosal innate immune system. Environ. Sci. Eur..

[17] McLellan N.L., Manderville R.A. (2017). Toxic mechanisms of microcystins in mammals. Toxicol. Res..

[18] Svirčev Z., Lalić D., Savić G.B., Tokodi N., Backović D.D., Chen L., Meriluoto J., Codd G.A. (2019). Global geographical and historical overview of cyanotoxin distribution and cyanobacterial poisonings. Arch. Toxicol..

[19] Nobre A.C.L., Martins A.M.C., Havt A., Benevides C., Lima A.A.M., Fonteles M.C., Monteiro H.S.A. (2003). Renal effects of supernatant from rat peritoneal macrophages activated by microcystin-LR: Role protein mediators. Toxicon.

[20] Khan S.A., Ghosh S., Wickstrom M., Miller L.A., Hess R., Haschek W.M., Beasley V.R. (1995). Comparative pathology of microcystin-lr in cultured hepatocytes, fibroblasts, and renal epithelial cells. Nat. Toxins.

[21] Fischer W., Dietrich D. (2000). Pathological and biochemical characterization of microcystin-induced hepatopancreas and kidney damage in carp (*Cyprinus carpio*). Toxicol. Appl. Pharmacol..

[22] Van der Merwe D., Sebbag L., Nietfeld J.C., Aubel M.T., Foss A., Carney E. (2012). Investigation of a *Microcystis aeruginosa* cyanobacterial freshwater harmful algal bloom associated with acute microcystin toxicosis in a dog. J. Vet. Diagn. Investig..

[23] Wang Z., Li G., Wu Q., Liu C., Shen J., Yan W. (2019). Microcystin-LR exposure induced nephrotoxicity by triggering apoptosis in female zebrafish. Chemosphere.

[24] Moreno I., Pichardo S., Josa A., Gómez-Amores L., Mate A., Vazquez C.M., Cameán A.M. (2005). Antioxidant enzyme activity and lipid peroxidation in liver and kidney of rats exposed to microcystin-LR administered intraperitoneally. Toxicon.

[25] Milutinović A., Sedmak B., Horvat-Znidarsic I., Suput D. (2002). Renal injuries induced by chronic intoxication with microcystins. Cell. Mol. Biol. Lett..

[26] Qin W., Xu L., Zhang X., Wang Y., Meng X., Miao A., Yang L. (2010). Endoplasmic reticulum stress in murine liver and kidney exposed to microcystin-LR. Toxicon.

[27] Jayaraj R., Rao P.L. (2006). Protein phosphorylation profile and adduct formation in liver and kidney of microcystin-LR-treated mice. Toxicon.

[28] Al-Jassabi S., Ahmed K.-A., Abdulla M. (2012). Antioxidant effect of curcumin against microcystin-LR-induced renal oxidative damage in Balb/c mice. Trop. J. Pharm. Res..

[29] Xu C., Shu W.-Q., Cao J., Qiu Z.-Q., Zhao Q., Chen J.-A., Zeng H., Fu W.-J. (2007). [Antagonism effects of green tea against microcystin induced oxidant damage on liver and kidney]. Zhonghua Yu Fang Yi Xue Za Zhi.

[30] Yi X., Xu S., Huang F., Wen C., Zheng S., Feng H., Guo J., Chen J., Feng X., Yang A.F. (2019). Effects of chronic exposure to microcystin-LR on kidney in mice. Int. J. Environ. Res. Public Health.

[31] Berl T., Combs S., Kimmel P.L., Rosenberg M.E. (2015). Chapter 30—Water metabolism in chronic kidney disease. Chronic Renal Disease.

[32] Roué M., Darius H.T., Chinain M. (2018). Solid Phase Adsorption Toxin Tracking (SPATT) technology for the monitoring of aquatic toxins: A review. Toxins.

[33] Gupta N., Pant S.C., Vijayaraghavan R., Rao P.V.L. (2003). Comparative toxicity evaluation of cyanobacterial cyclic peptide toxin MC variants (LR, RR, YR) in mice. Toxicology..

[34] Miao H.-F., Qin F., Tao G.-J., Tao W.-Y., Ruan W.-Q. (2010). Detoxification and degradation of microcystin-LR and -RR by ozonation. Chemosphere.

[35] Díez-Quijada L., Puerto M., Gutiérrez-Praena D., Llana-Ruiz-Cabello M., Jos A., Cameán A.M. (2019). Microcystin-RR: Occurrence, content in water and food and toxicological studies. A review. Environ. Res..

[36] Wu S., Wang S., Yang H., Xie P., Ni L., Xu J. (2008). Field studies on the environmental factors in controlling microcystin production in the subtropical shallow lakes of the Yangtze River. Bull. Environ. Contam. Toxicol..

[37] Zhang S.H., Chang J.J., Cao J.Y., Yang C.L. (2014). Comparative studies on growth and physiological responses of unicellular and colonial *Microcystis aeruginosa* to *Acorus calamus*. Bull. Environ. Contam. Toxicol..

[38] Zhang Y., Huang X., Xiao W., Zhong Q., Gu K. (2013). Purification and identification of microcystin-RR. Chin. J. Chromatogr..

[39] Ding W.-X., Ong C.N. (2003). Role of oxidative stress and mitochondrial changes in cyanobacteria-induced apoptosis and hepatotoxicity. FEMS Microbiol. Lett..

[40] Huang P., Zheng Q., Xu L.-H. (2010). The apoptotic effect of oral administration of microcystin-RR on mice liver. Environ. Toxicol..

[41] Sedan D., Laguens M., Copparoni G., Aranda J.O., Giannuzzi L., Marra C.A., Andrinolo D. (2015). Hepatic and intestine alterations in mice after prolonged exposure to low oral doses of Microcystin-LR. Toxicon.

[42] Mrdjen I., Morse M.A., Ruch R.J., Knobloch T.J., Choudhary S., Weghorst C.M., Lee J. (2018). Impact of Microcystin-LR on liver function varies by dose and sex in mice. Toxins.

[43] Fischer W., Altheimer S., Cattori V., Meier P., Dietrich D., Hagenbuch B. (2005). Organic anion transporting polypeptides expressed in liver and brain mediate uptake of microcystin. Toxicol. Appl. Pharmacol..

[44] Zhao Y., Xie P., Tang R., Zhang X., Li L., Li D. (2008). In vivo studies on the toxic effects of microcystins on mitochondrial electron transport chain and ion regulation in liver and heart of rabbit. Comp. Biochem. Physiol. C Toxicol. Pharmacol..

[45] Žegura B., Gajski G., Straser A., Vrhovac V.G., Filipic M. (2011). Microcystin-LR induced DNA damage in human peripheral blood lymphocytes. Mutat. Res. Toxicol. Environ. Mutagen..

[46] Feher J., Feher J. (2017). Chapter 7.4—Tubular reabsorption and secretion. Quantitative Human Physiology.

[47] Lowe J., Souza-Menezes J., Freire D.S., Mattos L.J., Castiglione R.C., Barbosa C.M., Santiago L., Ferrão F.M., Cardoso L.H.D., da Silva R.T. (2012). Single sublethal dose of microcystin-LR is responsible for different alterations in biochemical, histological and physiological renal parameters. Toxicon.

[48] Ding J., Wang J., Xiang Z., Diao W., Su M., Shi W., Wan T., Han X. (2017). The organic anion transporting polypeptide 1a5 is a pivotal transporter for the uptake of microcystin-LR by gonadotropin-releasing hormone neurons. Aquat. Toxicol..

[49] Feurstein D., Holst K., Fischer A., Dietrich D. (2009). Oatp-associated uptake and toxicity of microcystins in primary murine whole brain cells. Toxicol. Appl. Pharmacol..

[50] Fischer A., Hoeger S., Stemmer K., Feurstein D., Knobeloch D., Nussler A., Dietrich D. (2010). The role of organic anion transporting polypeptides (OATPs/SLCOs) in the toxicity of different microcystin congeners in vitro: A comparison of primary human hepatocytes and OATP-transfected HEK293 cells. Toxicol. Appl. Pharmacol..

[51] Kusuhara H., Sugiyama Y. (2002). Role of transporters in the tissue-selective distribution and elimination of drugs: Transporters in the liver, small intestine, brain and kidney. J. Control. Release.

[52] Kim R.B. (2003). Organic anion-transporting polypeptide (OATP) transporter family and drug disposition. Eur. J. Clin. Investig..

[53] Mikkaichi T., Suzuki T., Tanemoto M., Ito S., Abe T. (2004). The Organic Anion Transporter (OATP) family. Drug Metab. Pharmacokinet..

[54] Cheng X., Maher J., Chen C., Klaassen C.D. (2005). Tissue distribution and ontogeny of mouse organic anion transporting polypeptides (OATPS). Drug Metab. Dispos..

[55] Cheng X., Klaassen C.D. (2009). Tissue distribution, ontogeny, and hormonal regulation of xenobiotic transporters in mouse kidneys. Drug Metab. Dispos..

[56] Hooser S.B., Beasley V.R., Lovell R.A., Carmichael W.W., Haschek W.M. (1989). Toxicity of microcystin LR, a cyclic heptapeptide hepatotoxin from microcystis aeruginosa, to rats and mice. Vet. Pathol..

[57] Carey R.M., Padia S.H., Singh A.K., Williams G.H. (2018). Chapter 1—Physiology and regulation of the renin–angiotensin–aldosterone system. Textbook of Nephro-Endocrinology.

[58] Zhong Q., Sun F., Wang W., Xiao W., Zhao X., Gu K. (2017). Water metabolism dysfunction via renin-angiotensin system activation caused by liver damage in mice treated with microcystin-RR. Toxicol. Lett..

[59] Zhao Y., Xue Q., Su X., Xie L., Yan Y., Steinman A.D. (2015). Microcystin-LR induced thyroid dysfunction and metabolic disorders in mice. Toxicology.

[60] Dabrowski E., Kadakia R., Zimmerman D. (2016). Diabetes insipidus in infants and children. Best Pract. Res. Clin. Endocrinol. Metab..

[61] Kopp P.A. (2016). Preface—Disturbances of water and electrolyte balance. Best Pract. Res. Clin. Endocrinol. Metab..

