# Supplementary material for: Differences in Abnormal Water Metabolism between SD Rats and KM Mice Intoxicated by Microcystin-RR
Source: Int J Environ Res Public Health. 2021 Feb 16;18(4):1900. doi: 10.3390/ijerph18041900 (PMC7920292; doi:10.3390/ijerph18041900)
Supplement: Supplementary file 1 [file ijerph-18-01900-s001.pdf]

International Journal of
Environmental Research and Public Health

MDPI

Supplementary material

The MC-RR used in this study was extracted and purified from natural water blooms. The purified MC-RR was qualitative and quantitative by MC-RR standard (Enzo Life Sciences, Inc., Switzerland) through the high performance liquid chromatography (HPLC).

A Waters 2695 HPLC system coupled to a Photo-Diode Array Waters 2996 detector and equipped with a Zorbax SB-C18 column (250 mm×4.6 mm i.d., 5 μm particle sizes) was used for MC-RR analysis. Working conditions: absorption spectrum was from 200 nm to 800 nm, the mobile phase was 35% water (with 0.05% [vol/vol] trifluoroacetic acid) : 65% methanol, flow rate was 1 mL/min, the column temperature was kept at 40 °C, and the injection volume was 10 μL.

The chromatograms are shown in Figure S1.

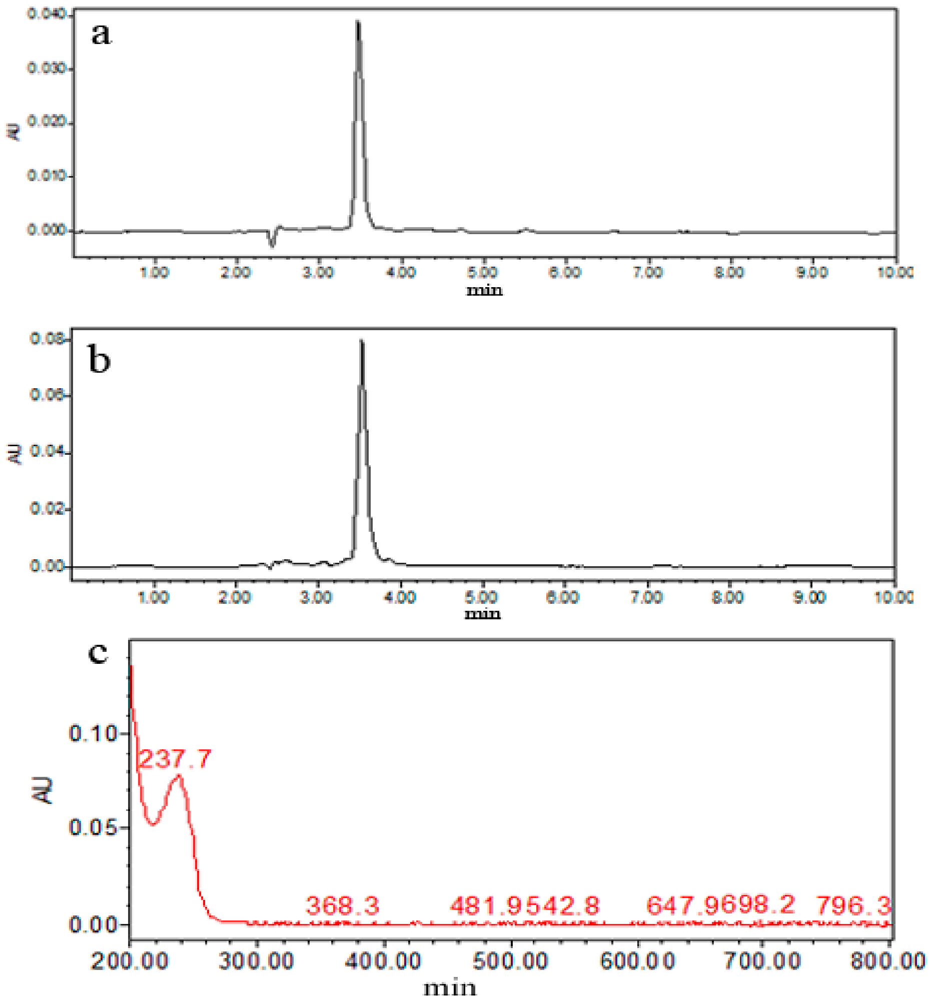

Figure S1. Chromatograms of MC-RR standard (a) and the purified MC- RR (b and c).
